# A case of multisystem inflammatory syndrome in children: A case report

**DOI:** 10.1097/MD.0000000000036329

**Published:** 2023-12-01

**Authors:** Junsheng Jiang, Zheng Fan, Yueyan Mao

**Affiliations:** a Department of Pediatrics, Linping Branch of the Second Affiliated Hospital of Zhejiang University, Hangzhou, China.

**Keywords:** case report, child, Kawasaki disease, multisystem inflammatory syndrome

## Abstract

**Rationale:**

Multisystemic inflammatory syndrome is a syndrome of multisystem involvement caused by a novel coronavirus infection that can lead to cardiogenic shock and death in children.

**Patient concerns:**

A 4-year-old girl was diagnosed with multiple organ and multiple system involvement after Kawasaki disease.

**Diagnosis:**

Novel coronavirus infection-associated multisystem inflammatory syndrome in children was considered.

**Interventions:**

The patients received aspirin, methylprednisolone and gammaglobulin to treat multisystem inflammatory syndrome.

**Outcomes:**

After treatment, the child recovered and was discharged from the hospital.

**Lessons:**

Multisystem inflammatory syndrome is often mistaken for Kawasaki disease, fortunately, their treatments are similar, the purpose of this case is to remind clinicians of the need for early management of children with multisystem failure following novel coronavirus infection, increase the detection rate, and save the life of the child.

## 1. Introduction

Multisystemic inflammatory syndrome in children has been reported during the global epidemic of novel coronavirus infections, but it is rare in Asian populations.^[1–3]^ In children, multisystemic inflammatory syndrome (MIS) can be cumulative and multi-systemic, and common clinical signs include high fever, enlarged lymph nodes, polymorphic rash, conjunctivitis, and coronary artery dilatation.^[2,4–6]^ In this case report, we describe a 4-year-old girl who had a multisystemic inflammatory syndrome that was misdiagnosed as Kawasaki disease.

## 2. Case presentation

The child, a female, was admitted to the hospital with a fever for 4 days, and a rash for 3 days, The rash gradually increased, and during the course of the disease, the child was depressed, had a poor appetite, significant abdominal pain, and normal bowel movements. Medical, family and psychosocial history including relevant genetic information are normal. Physical examination: temperature 38.ºC, pulse 126/min, respiration 32/min, blood pressure 87/52 mm Hg, the child limbs and trunk can be seen in a large rash, bilateral neck can be palpable swollen lymph nodes, bulbar conjunctiva congestion, poplar tongue, swelling of the hands and feet, extremity end warmth. Complementary check: Leukocytes:13.43*10^^9^, lymphocytes:7.5%, neutrophils:85.2%, platelets:89*10^^9^, C-reactive protein:139 mg/L. Erythrocyte sedimentation rate:42 mm/1H, D-dimer:5.05 mg/L, IL-6:299, B-type natriuretic peptide:1887 pg/mL, Novel Coronavirus Nucleic Acid Positive, ferritin:7301 ng/mL, Glutamic aminotransferase: 995 U/L, aspartate aminotransferase: 240 U/L, Abdominal color ultrasonography showed no abnormality in liver, gallbladder, spleen and pancreas. No abnormality was found in the reexamination before discharge. Chest computed tomography showed inflammatory lesions in the right middle lobe and the left lower lobe of the lung. Chest CT examination before discharge indicated that double pneumonia was more obviously absorbed than before. No abnormalities in the coronary arteries of the heart.

## 3. Treatment

Kawasaki disease was considered on admission and treated with symptomatic supportive therapy and Fasting, gastrointestinal decompression, omeprazole acid inhibition, piperacillin-tazobactam anti-bacterial infection, dopamine to improve circulation, and rehydration treatment. On the second day, the diagnosis of novel coronavirus-associated multisystemic inflammatory syndrome in children was revised by combining the child clinical manifestations and various examination results. After intravenous gammaglobulin, oral aspirin, and methylprednisolone, the child temperature normalized and the rash disappeared. A review of routine blood leukocytes and C-reactive protein decreased to normal, the child condition was stable, and the child was discharged from the hospital with aspirin low-dose oral therapy. The child was regularly followed up with no abnormalities on cardiac ultrasound and blood tests after discharge.

## 4. Discussion

As MIS in children is a newly discovered disease associated with novel coronavirus infection, its long-term prognosis is unclear, but there is a possibility of concomitant cardiac insufficiency.^[4,6]^ The US Centers for Disease Control and Prevention, the Royal College of Pediatrics and Child Health, and the World Health Organization have proposed different diagnostic criteria for MIS in children in terms of age of onset, clinical features, laboratory tests associated with inflammation, and evidence of novel coronavirus infection.^[7]^ The different diagnostic criteria differ in terms of age and fever requirements, but there is consensus on multisystem damage, elevated inflammatory markers, exclusion of other pathogens causing the disease, and evidence supporting the association with novel coronavirus infection. In this case, the first manifestation of the disease was fever for 4 days and rash for 3 days. There was multisystem involvement of the skin and mucous membranes, gastrointestinal, respiratory, and cardiovascular systems. Laboratory tests showed neutrophilia, lymphocytopenia, erythrocyte sedimentation rate of 44 mm/1 hour, and D-dimer of 5.05 mg/L. The child and his parents were positive for novel coronavirus antigen. The above clinical manifestations and laboratory findings were consistent with the diagnostic criteria for MIS in children. The clinical manifestations of childhood MIS are similar to those of Kawasaki disease, and there is no evidence that childhood MIS is a specific type of Kawasaki disease. Comparing childhood MIS with Kawasaki disease, childhood MIS is more common in older children and adolescents, while Kawasaki disease occurs more often in infants and young children.^[4,6]^ Although both childhood MIS and Kawasaki disease have clinical manifestations of multiorgan system involvement, gastrointestinal system involvement is common in childhood MIS, whereas children with Kawasaki disease rarely have gastrointestinal symptoms.^[8]^ Decreased left ventricular ejection function is common as a complication of childhood MIS, but coronary artery disease is also seen.^[9]^ In this case, the child presented with fever, polymorphic rash, prune tongue, mucosal involvement of the mouth and lips, and enlarged cervical lymph nodes on imaging, which could be easily confused with Kawasaki disease at the time of diagnosis. However, the presence of digestive system involvement, which is rare in Kawasaki disease, and a history of exposure to novel coronavirus and evidence of infection excluded Kawasaki disease, and the diagnosis was revised to MIS in children during treatment. Fortunately, the child was cured and discharged. However, this is only a single case. For this kind of patients with a novel coronavirus infection, the cause of the occurrence, and the dosage of postoperative drugs have not yet been determined, and more cases of multi-center studies are needed to make an objective evaluation.

## 5. Conclusion

In the case of a child with MIS, gammaglobulin, hormones, and aspirin treatment are required, this may reduce the possibility of coronary artery damage.

## Author contributions

**Methodology:** Zheng Fan.

**Supervision:** Yueyan Mao.

**Writing – original draft:** Junsheng Jiang.
